# Editorial: Metabolism As a Therapeutic Target

**DOI:** 10.3389/fonc.2017.00266

**Published:** 2017-11-09

**Authors:** Amedeo Columbano, Silvia Giordano

**Affiliations:** ^1^Unit of Oncology and Molecular Pathology, Department of Biomedical Sciences, Università degli studi di Cagliari, Cagliari, Italy; ^2^Department of Oncology, Università di Torino, Candiolo Cancer Institute-FPO, IRCCS, Candiolo, Italy

**Keywords:** cancer metabolic reprogramming, pentose phosphate pathway, epithelial–mesenchymal transition, autophagy, TRAP1, obesity

**Editorial on the Research Topic**

**Metabolism As a Therapeutic Target**

Metabolic reprogramming, also known as “Warburg effect,” is considered a distinct hallmark of cancer, although its relevance appears to be even more general, as all the identified hallmarks rely on metabolic changes. Indeed, decreased activity of Phase I enzymes, paralleled by enhancement of Phase II ones, is required for conferring resistance to ROS or chemically induced toxicity; metabolic enzymes are required to initiate vessel sprouting during angiogenesis; modifications of the autophagic apparatus eliminate defective organelles to reduce oxidative stress and enable tumor cells to cope with their high metabolic demand. Support for metabolism involvement in cancer also comes from the identification of germline mutations in metabolic genes of patients affected by hereditary cancers.

Although the precise mechanisms responsible for the complex rewiring of metabolic circuitries remain poorly understood, it has become clear that they can be exploited as an Achilles’ heel for therapeutic targeting. Preclinical and clinical evidence, indeed, suggests that metabolism-targeting drugs can efficiently interfere with tumor progression. New strategies combining metabolic drugs with chemotherapy or targeted therapies are now under evaluation, with the hope that they can help overcome resistance onset, at the moment the main cause of therapeutic failure.

As this is a recent and rapidly evolving field, with several aspects often controversial, the present research topic focuses on current achievements and future perspectives.

Masgras et al. reviewed our present knowledge of the mitochondrial molecular chaperone TRAP1, a key regulator of mitochondrial bioenergetics in tumor cells that impacts neoplastic growth by downregulating both respiratory complex IV and succinate dehydrogenase (SDH). SDH inhibition causes accumulation of succinate and activation of the transcription factor HIF-1α, which promotes glutamine and glucose utilization. In their review, the authors describe the complexity of TRAP1 effects on tumor cells and the possibility of its interaction with other client proteins in cells. In this scenario, the possibility of connecting TRAP1 chaperone activity with oncogenic transduction pathways might elucidate the tumor type and the stage at which the metabolic rewiring prompted by TRAP1 is pivotal for neoplastic progression.

Kowalik et al. illustrated the therapeutic potential hidden in the Pentose Phosphate Pathway and underlined that such potential is strongly limited by the lack of specific pharmacological inhibitors. Nevertheless, the evidence that increased hepatocyte glucose-6-phosphate dehydrogenase (G6PD) expression is an early event in animal models of hepatocarcinogenesis, is a feature unique to the tumorigenic process and discriminates human hepatocellular carcinoma (HCC) from peritumoral tissue, makes the search for reliable G6PD inhibitors very attractive in the HCC field. Indeed, the decreased NADPH generation upon inhibition of the oxidative PPP could (i) selectively eradicate cancer cells by decreasing their resistance to high intracellular ROS levels; (ii) increase, in conjunction with already-approved therapies (i.e., kinases inhibitors or other chemotherapeutic agents), the susceptibility of cancer cells to anticancer drugs. The design of specific inhibitors targeting PPP might thus represent a useful therapeutic tool, in particular, for HCC.

Activation of PPP has recently been suggested to be one of the mechanisms by which deregulated NRF2-KEAP1 signaling promotes cell proliferation and tumorigenesis. The work by Taguchi and Yamamoto illustrates the Gianus behavior of NRF2. Both NRF2 inducers and NRF2 inhibitors are expected to function as anticancer drugs: while NRF2 inducers protect normal cells from carcinogens, NRF2 inhibitors suppress the proliferation of cancer cells displaying aberrant NRF2 activation. However, many questions related to NRF2 inducers and inhibitors must be answered before they can be applied in anticancer therapy. An emerging possibility is to use NRF2 inducers for cancer therapy in combination with conventional anticancer agents. In this regard, there is a concern whether long-term application of NRF2 inducers may eventually support the switch of cryptic cancer-initiating cells into real cancer cells.

Perturbation of endoplasmic reticulum (ER) homeostasis results in “ER stress,” determining the activation of a program defined as unfolded protein response (UPR), whose primary aim is to restore the physiological activity of these organelles. As underlined by Corazzari et al., although the UPR program is primarily a pro-survival process, sustained stress may results in cell death. In their review, Corazzari et al. discuss the role played by the UPR program in tumor initiation, progression, and resistance to therapy, highlighting the molecular mechanisms that regulate the survival/death switch.

The review by Morandi et al. investigates the metabolic rewiring that takes place in tumor cells undergoing epithelial-to-mesenchymal transition (EMT). EMT execution is sustained by the metabolic requirements of a fast-growing tumor, aiming at overcoming nutrients and oxygen supply limitation and colonizing secondary sites to secure the adequate support of energy and nutrients. Independently of the stimuli-inducing EMT, the key event is the loss of E-cadherin, whose expression is regulated by transcription factors such as Snail1, Slug, Twist, and ZEB1. However, increasing evidence links metabolic deregulation to the EMT program. Morandi et al. gathers the recent findings on EMT and metabolic reprogramming in cancer and discusses how targeting certain metabolic pathways/hubs may impact on EMT and thus on cancer progression.

Finally, the article by Doerstling et al. summarizes the interplay between obesity and cancer metabolism. High serum levels of insulin and insulin-like growth factor 1, adipose tissue dysfunction, and nutrient-replete circulation are some of the mechanisms by which obesity supports malignant cell growth. The relationship between obesity and cancer is strengthened by the observation that by attenuating obesity-associated signaling pathways and activating nutrient stress responses, calorie restriction regimens reduce obesity-associated cancer risk. The adipose tissue is also characterized by high abundance of macrophages and other immune cells that increase expression of pro-inflammatory cytokines by adipocytes. In consequence, circulating levels of cytokines are quite high in overweight individuals. Notably, a highly significant epidemiologic association has been identified between high levels of circulating cytokines and cancer risk.

Overall, many aspects of cancer metabolism are still poorly known. The recently acquired knowledge that metabolic alterations sustain almost all the known hallmarks of cancer points at metabolism as a very interesting therapeutic target. Even though few specific drugs are currently available, it is expected that their number will rapidly increase, opening new therapeutic perspectives, likely in association with drugs targeting tumor drivers.

## Author Contributions

The two authors are the editors of the research topic on “Metabolism as a therapeutic target.”

## Conflict of Interest Statement

The authors declare that the research was conducted in the absence of any commercial or financial relationships that could be construed as a potential conflict of interest.

